# Accuracy of Endoscopic Ultrasonography for Determining the Treatment Method for Early Gastric Cancer

**DOI:** 10.1155/2012/245390

**Published:** 2012-11-20

**Authors:** Koichiro Mandai, Kenjiro Yasuda

**Affiliations:** Department of Gastroenterology, Kyoto Second Red Cross Hospital, 355-5 Haruobi-cho, Kamigyo-ku, Kyoto 602-8026, Japan

## Abstract

*Background*. Endoscopic resection (ER) for early gastric cancer (EGC) is a minimally invasive and curative treatment. The value of endoscopic ultrasonography (EUS) in determining the therapeutic strategy for EGC was assessed in this study. *Materials and Methods*. Pretreatment EUS was performed on 406 EGCs. The lesions were divided into the histological categories m/sm1 and sm2. The EUS-determined depths of invasion were classified as EUS-M/SM1, EUS-SM2, and EUS-MP or deeper. An analysis of the factors influencing the EUS-based depth determination was then conducted. *Results*. Most (92.8%) of the EUS-M/SM1 group belonged to the m/sm1 histological category. Ulcerated lesions, tumor size of larger than 2 cm, and the use of an ultrasound endoscope were independently associated with misdiagnosis of the depth of EGC by EUS. The ulcerated lesions had a significantly higher probability of overestimation. *Conclusions*. EUS is a useful method for determining the therapeutic strategy for EGC. Special attention should be paid not to overestimate the depth of cancer invasion when determining the ulcerated lesions and the type of curative procedure to be used.

## 1. Introduction

Endoscopic resection (ER) for the treatment of early gastric cancer (EGC) is currently accepted as a minimally invasive and curative treatment. According to the *Japanese Gastric Cancer Treatment Guidelines *[1], the indication for ER is a mucosal lesion, less than 2 cm in size, without ulceration. However, the guidelines have also expanded the indications for ER into the following categories that have very low possibilities of lymph node metastasis [1, 2]: (1) differentiated, mucosal cancer lesions, larger than 2 cm, without ulcerative findings [UL(−)]; (2) differentiated, mucosal cancer lesions ≤3 cm in size, with ulcerative findings [UL(+)]; (3) undifferentiated, mucosal cancer lesions ≤2 cm, UL(−); (4) differentiated lesions ≤3 cm in size, with submucosal invasion of less than 500 *μ*m (sm1). Therefore, accurate determination of the depth of gastric cancer invasion is increasingly important to the determination of the therapeutic strategy.

Endoscopic ultrasonography (EUS) is one of the diagnostic methods for determining the depth of gastric cancer invasion. In this study, EUS was evaluated for its utility in determining the depth of gastric cancer invasion, and for its necessity in determining therapeutic strategy.

## 2. Materials and Methods

Pretreatment EUS was performed on 406 EGCs with histologically proven mucosal and submucosal cancer lesions between January 2006 and December 2009 at Kyoto Second Red Cross Hospital (Kyoto, Japan). Endoscopic mucosal resection (EMR) was performed on 18 lesions; endoscopic submucosal dissection (ESD) was performed on 202; 186 lesions were treated surgically. The results of these treatments were retrospectively reviewed. 

Based on the classification system of the Japanese Gastric Cancer Association [3], the locations of the stomach lesions were divided into the upper, middle, and lower thirds of the stomach; each lesion was classified as either differentiated or undifferentiated, based on a histological assessment. The macroscopic features of the lesions were diagnosed by endoscopic findings and were classified as elevated type (0-I and 0-IIa), UL(−) type (0-IIb and 0-IIc without ulcerative findings), and UL(+) type (0-IIc with ulcerative findings, and 0-III) according to the classifications described in Table 1 and based on whether or not they were ulcerated. 

The depth of cancerous invasion was histologically classified as follows: lesion confined to mucosal layer (m); <500 *μ*m invasion into the submucosal layer (sm1); >500 *μ*m deep invasion into the submucosal layer (sm2). The lesions were divided into the histological m/sm1 group, for which ER may be suitable, and the sm2 group, for which surgery was indicated. 

**Figure 1:**
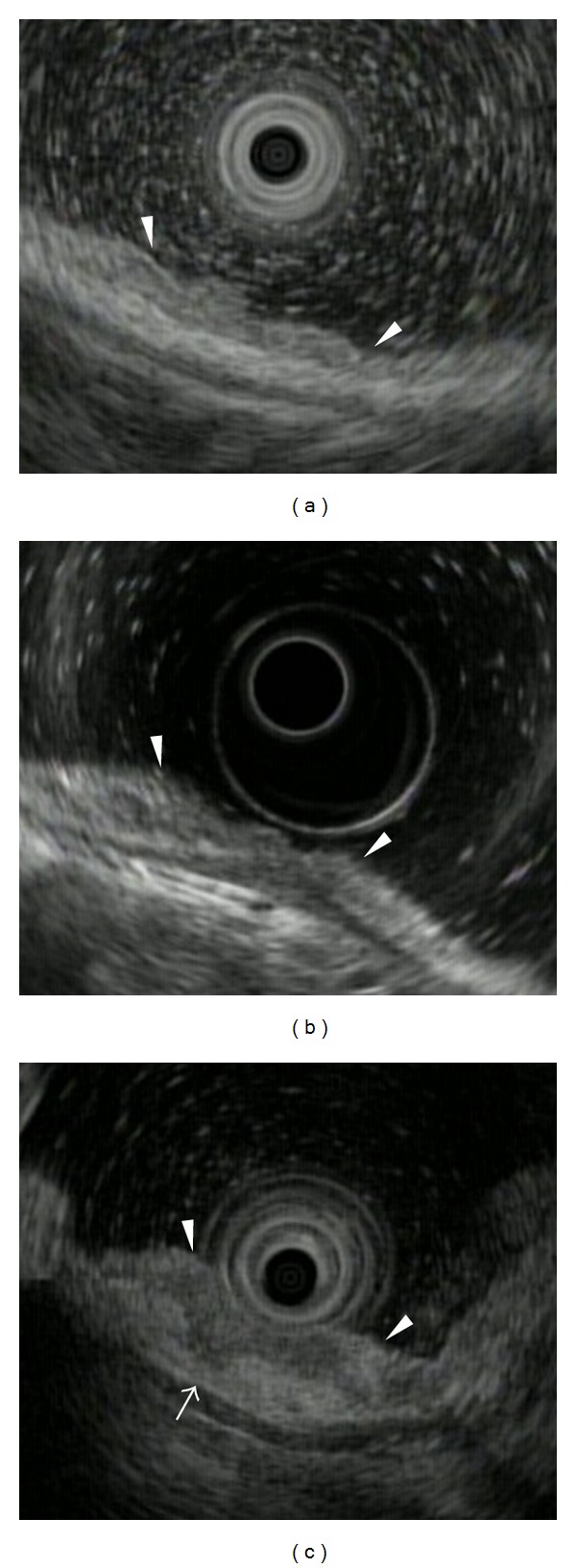
Endoscopic ultrasonography images of early gastric cancer. (a) EUS-M/SM1, type 0-IIc: there is no destruction in the first and second layers. The third layer looks normal. (b) EUS-M/SM1, type 0-IIc + III: the third layer shows smooth tapering and convergence. (c) EUS-SM2, type 0-IIa: a hypoechoic tumor shows the submucosal invasion. (white arrow).

EUS was used to determine the depth of cancer invasion. Two EUS devices were used: the ultrasound probe (US-probe; UM-3R, 20 MHz, Olympus, Tokyo, Japan) was selected for use with smaller or flat lesions, and the ultrasound endoscope (US-endoscope; GF-UM-2000, Olympus) was selected for use with larger or deep, depressed lesions. The EUS-determined depths of invasion were classified as lesions with no abnormality in the submucosal layer or smooth tapering of the submucosal layer (EUS-M/SM1) (Figures 1(a) and 1(b)); lesions with irregularity of the submucosal layer (EUS-SM2) (Figure 1(c)); lesions with an abrupt interruption of the submucosal or deeper layer (EUS-MP or deeper).

The location, macroscopic features, tumor size, histological type, and EUS type were analyzed to determine if they influenced the EUS diagnosis of the depth of cancer invasion. “Dr. SPSS II for Windows” was used for statistical analysis. A Chi-square test was used for the univariate analyses, and logistic regression was used for multivariate analyses.

## 3. Results and Discussion

### 3.1. Accuracy of EUS and Risk Factors for Misdiagnosis of the Depth of Cancer Invasion

Of the 406 lesions evaluated, 52 were located in the upper third of the stomach; 45, in the middle third; 309, in the lower third. Morphologically, 152 lesions were classified as the elevated type; 171, the UL(−) type; 83 as the UL(+) type. Histologically, 314 lesions were the differentiated type and 92 lesions were the undifferentiated type. The US-probe was used to evaluate 298 lesions, and the US-endoscope was used in the remaining 108 lesions. 

Previous reports have indicated that depth determination accuracy, by EUS, in EGC may range from 67%–90% [4–9]. In this study, when the lesions were divided into the histological m/sm1 and sm2 categories, the overall diagnostic accuracy of EUS was 74.6% (303/406) (Table 2). 

Depending on the macroscopic features, the tumor size, and the histological type, the accuracy of EUS varied widely. The accuracy also varied depending on the ultrasound instrument that was used, with the US-probe and US-endoscope having accuracies of 85.2% and 45.3%, respectively. The univariate analysis showed that the accuracy was significantly lower for the UL(+), the tumor size of larger than 2 cm, and the undifferentiated types of lesions as well as for those diagnosed with the US-endoscope (Table 3). Multivariate analysis of these 4 factors showed that the UL(+) type (OR 8.573; 95% CI 4.632–15.867), the tumor size of larger than 2 cm (OR 2.071; 95% CI 1.149–3.731), and the use of US-endoscope (OR 2.472; 95% CI 1.330–4.593) were independently associated with misdiagnosis of the depth of EGC by EUS (Table 4).

In these risk factors for misdiagnosis, the UL(+) type and the use of US-endoscope had a significantly higher probability of overestimation (Table 5). 

According to previous reports, lesions with ulcerous changes [8, 10] or lesions of the depressed [6, 11] or undifferentiated types [6, 9] or tumor size of larger than 3 cm [9] or lesions located in the upper third of the stomach [5, 11] were associated with incorrect depth determinations by EUS. These reported results are similar to our study.

Another report showed that the accuracy of US-probe was significantly higher than that of US-endoscope [4]. In our study, one of the risk factors for misdiagnosis of the depth of EGC was also associated with the use of the US-endoscope. The US-probe is particularly suitable for the determination of the depth of EGC because the frequency of the US-probe is higher than that of the US-endoscope, allowing the US-probe to have a higher resolution within the shallower layers. However, the selection of the type of ultrasound instrument used to make the depth determination was based on the endoscopic appearance of the tumor, such as its size, height of elevation, and depth of depression. The US-probe was used for smaller lesions or lesions with shallower depressions that were easy to diagnose as mucosal cancer, whereas the US-endoscope was used for lesions with a deep ulceration that were difficult to distinguish between a benign fibrosis and a cancerous invasion. This selection bias could explain why the accuracy of the US-probe was higher than that of the US-endoscope. 

### 3.2. Therapeutic Strategy of EGC

In this study, most of the EUS-M/SM1 group lesions belonged to the histological m/sm1 category (Table 2: 92.8%, 260/280). Therefore, ER is appropriate for lesions determined to be EUS-M/SM1. On the other hand, the EUS-SM2 group included many histological m/sm1 lesions (Table 2: 56.5%, 56/99) for which ER, especially ESD, might be a curative treatment. However, most of these lesions have ulcerative changes (Table 6) which are predictive of difficult dissections during ESD. Therefore, although ESD might be considered for lesions determined to be EUS-SM2 or deeper, surgery is also an appropriate treatment for these lesions.

## 4. Conclusions

EUS is a useful tool for determining the therapeutic strategy for EGCs. However, EUS is not the best method to correctly determine the depth of the EGC invasion, in the cases of UL(+) lesions or tumor size of larger than 2 cm. Special attention should be paid not to overestimate the depth of cancer invasion when determining the UL(+) lesions and the type of curative procedure to be used. ER should be performed for lesions classified as EUS-M/SM1, whereas surgery is an appropriate treatment for EUS-SM2 lesions.

## Figures and Tables

**Table 1:** Macroscopic classification of early gastric cancers [3].

Type 0-I (protruding)	Polypoid tumors
Type 0-IIa (superficial elevated)	Slightly elevated tumors
Type 0-IIb (superficial flat)	Tumors without elevation or depression
Type 0-IIc (superficial depressed)	Slightly depressed tumors
Type 0-III (excavated)	Tumors with deep depression

**Table 2:** Accuracy of cancer invasion depth as determined by endoscopic ultrasound.

Histology	EUS				
	M/SM1	SM2	MP deeper	Total	Accuracy
m/sm1	**260**	56	11	327	79.5% (260/327)
sm2	20	**43**	16	79	54.4% (43/79)
PPV	92.8% (260/280)	43.4% (43/99)	—	—	

EUS: endoscopic ultrasonography; PPV: positive predictive value; M/SM1, SM2, and MP deeper are classifications of the depth of tumor invasion into the submucosa (see text for full description).

**Table 3:** Univariate analysis of factors affecting accuracy of determinations of the depth of cancer invasion.

	Correct (*n*)	Incorrect (*n*)	Accuracy (%)	*P* value	Odds ratio	95% CI (%)
Stomach location				0.802		
Upper third	40	12	76.9%		1	
Middle third	32	13	71.1%	0.514	1.354	0.54–3.37
Lower third	231	78	74.7%	0.738	1.112	0.56–2.25

Macroscopic features				**<0.001**		
Elevated type	131	21	86.1%		1	
UL(−) type	148	23	86.5%	0.924	0.969	0.51–1.83
UL(+) type	24	59	28.9%	**<0.001**	**15.33**	**7.91–29.71**

Tumor size				**<0.001**		
≤2 cm	217	36	85.7%		1	
>2 cm, ≤3 cm	52	32	61.9%	**<0.001**	**3.709**	**2.11–6.52**
>3 cm	34	35	49.2%	**<0.001**	**6.205**	**3.44–11.18**

Histology						
Differentiated	254	60	80.8%		1	
Undifferentiated	49	43	53.2%	**<0.001**	**3.715**	**2.26–6.10**

EUS type						
US-probe	254	44	85.2%		1	
US-endoscope	49	59	45.3%	**<0.001**	**6.951**	**4.23–11.41**

UL(+): ulcerated; UL(−): nonulcerated; EUS: endoscopic ultrasonography; US: ultrasound; ER: endoscopic resection.

**Table 4:** Multivariate analysis of factors affecting accuracy of the determination of the depth of cancer invasion.

	*P* value	Odds ratio	95% CI (%)
UL(+) type	**P < 0.001**	**8.573**	**4.632–15.867**
Tumor size >2 cm	**P = 0.015**	**2.071**	**1.149–3.731**
Undifferentiated	*P* = 0.108	1.664	0.895–3.093
US-endoscope	**P = 0.004**	**2.472**	**1.330–4.593**

**Table 5:** The tendency of misdiagnosis in the risk factors for misdiagnosis of the depth of cancer invasion.

	Overestimation (*n*)	Underestimation (*n*)	*P* value	Odds ratio	95% CI (%)
Macroscopic features					
UL(+) type	56	3	**<0.001**	**11.753**	**3.17–43.57**
Non-UL(+) type	27	17			

Tumor size					
>2 cm	56	11	0.294	1.697	0.62–4.58
≤2 cm	27	9			

EUS type					
US-endoscope	55	4	**<0.001**	**7.858**	**2.39–25.73**
US-probe	28	16			

**Table 6:** Causes of m/sm1 cancer being classified as EUS-SM2 or deeper.

	67 lesions
Wrong evaluation of ulcerative change	44 (65.6%)
Presence of cystic change beneath the lesion	2 (3.0%)
Unknown	21 (31.3%)

m/sm1 refers to histologically determined depths and EUS-SM2 or deeper refers to depths of EGC invasion determined by EUS (see text for full description).
